# Stunting disparities and its associated factors among preschool children of employed and unemployed mothers in Gondar City: a comparative community-based cross-sectional study

**DOI:** 10.3389/fnut.2023.1172501

**Published:** 2023-09-06

**Authors:** Berhan Tekeba, Bethelihem Tigabu Tarekegn, Alebachew Ferede Zegeye, Amare Demsie Ayele

**Affiliations:** ^1^Department of Pediatrics and Child Health Nursing, University of Gondar, Gondar, Ethiopia; ^2^Department of Medical Nursing, University of Gondar, Gondar, Ethiopia

**Keywords:** employed, Gondar city, prevalence, preschool stunting, unemployed

## Abstract

**Introduction:**

A stunted child refers to a child who is too short for his/her age, which is the most common cause of morbidity and mortality in children under five in developing countries. Stunting in preschool children is caused by a multitude of socioeconomic and child-related factors, including the employment status of women. This study aimed to compare the prevalence and factors associated with stunting of preschool children among employed and unemployed mothers in Gondar city, Northwest Ethiopia, in 2021.

**Methods:**

From 30 February to 30 March 2021, a community-based comparative cross-sectional study was conducted among 770 preschool children of employed and unemployed mothers in Gondar city. A structured questionnaire-based interview with anthropometric measurements was used to collect data. A multi-stage sampling technique was used. Data were entered into EPI Info version 7.22 and transferred to Stata version 14 for further analysis. To identify factors associated with stunting, a binary logistic regression analysis was used. The presence of an association was declared based on a *p*-value of <0.05 and confidence intervals.

**Results:**

A total of 770 preschool children participated in the study. The overall prevalence of stunting among preschool children was 39.7% (95% CI: 36.3–43.2). The prevalence was higher among preschool children of employed mothers (42.6%) (95% CI: 37.6–47.5) than among unemployed mothers (36.7%) (95% CI: 32.0–41.7). Maternal age [AOR = 2.8, 95% CI: 1.26–6.34] and wealth status [AOR = 0.32, 95% CI: 0.18–0.57] were significantly associated with stunting among unemployed mothers, while family size [AOR = 7.19, 95% CI: 2.95–17.5], number of children under the age of five [AOR = 1.92, 95% CI: 1.12–3.29], and having a home servant [AOR = 0.126, 95% CI: 0.06–0.26] were associated with stunting of preschool children among employed mothers.

**Conclusion:**

Stunting is more common in preschool children of employed mothers than in those of unemployed mothers. As a result, interventions such as raising awareness among employed mothers to devote time and care to their children, as well as concerned bodies assisting women with preschool or under-five children, is required. The nutrition intervention should focus on encouraging dietary diversity to combat the existing nutrition-associated stunting in children. Similarly, further research on the difference between employed and unemployed mothers' child stunting status as well as an investigation of extra variables such as the number of hours worked by an employed mother is also recommended to upcoming researchers.

## Background

Stunting is defined as a height-to-age ratio that is more than two standard deviations lower than the World Health Organization's (WHO) child growth standard median. Children who are below minus three standard deviations (3 SD) are considered severely stunted (1). It is chronic undernutrition, which results from long-term exposure to limited nutrient supply and frequent infection (2).

Globally, stunting affected 144 million children under the age of five in 2019. More than half of all stunted children under the age of five resided in Asia (78.2 million), and two out of every five lived in Africa (58.5 million). More than 50 million of these people live in sub-Saharan Africa, including Ethiopia (2).

Despite a global drop in under-five malnutrition rates, the risk of malnutrition remains significant, and it is the leading cause of morbidity and mortality. Stunting is estimated to be responsible for 17% of the mortality load in children under the age of five (3). The burden is greater in Africa, particularly in sub-Saharan Africa. According to the Ethiopian Mini Demographic and Health Survey 2019 report, 37% of children under the age of five are short for their age or stunted (below −2 SD), and 12% are severely stunted (below −3 SD). The prevalence of stunting generally increases steadily with age, from 22% among children aged 6–8 months up to 44% of children aged 48–59 months. Notably, the highest proportion of stunting in children (45%) was observed at ages 24–35 months (4).

Parental socio-demographic, economic, cultural, environmental, and other health-related aspects are the most common causes of stunting among preschool children. Poverty, low parental education, low food consumption, poor feeding practices, inadequate breastfeeding, frequent infections, family size, and birth interval are among the primary determinants of stunting (5).

Preschool children require greater attention because they are in a fast-growing stage of life (6) and are primarily dependent on their mothers for all nutritional needs. Hence, it is suggested that the mother be the major caretaker during this period. As a result, they are highly vulnerable to malnutrition. Early childhood stunting could result in poor cognitive and behavioral development (5, 7), low school achievement, and a weakened immune system, which contributes to more than half of all infectious disease mortality in this age range (8–10).

Women's roles in the past have been constrained to housework and home activity in many societies, including Ethiopia. However, this status has since altered, and women have begun to pursue employment outside their homes (11). According to EDHS 2016, 48% of married women aged 15–49 were employed in the 12 months preceding the survey (12). This shows that the number of employed women is currently higher than this statistic.

Entering the labor force has both negative and positive consequences. On the one hand, it increases the family income and may give the women some economic independence. It also supports the family economy by allowing them to purchase more or better food, use more preventative health services, and obtain treatment for sick children, resulting in better nutritional and physical health (13). On the other hand, it also increases her workload and reduces the time that she spends with her child, including food preparation and healthcare visits, resulting in malnutrition (14).

Over the last several decades, the Ethiopian government has established comprehensive nutritional programs to enhance children's nutritional status (15). As a result, the government has made significant progress in reducing the burden of childhood stunting. However, 37% of children under the age of five remained stunted, indicating a very significant public health relevance when compared to the WHO criterion level (16).

It is critical to demonstrate the scale of stunting in order to address the negative repercussions of stunting in preschool children, particularly in connection with maternal employment. However, the majority of studies in the country only focused on children aged 6–24 months. Determining the extent of stunting among preschool children and the new students entering school in the near future might provide baseline evidence for a better estimation of functional consequences, including poor cognition. In Ethiopia, mothers' employment in relation to preschool child stunting is hardly ever examined; therefore, this study aimed to evaluate the impact of maternal work on preschool child stunting, which would provide information to the relevant authority.

## Methods

### Study design, period, and area

A community-based comparative cross-sectional study was conducted among preschool children of employed and unemployed women in Gondar town from 30 February to 30 March 2021. The study was conducted in Gondar city located in the Central Gondar Zone of Amhara Regional State, Northwest Ethiopia. The city is 748 km northwest of Addis Ababa, the capital of Ethiopia, and approximately 180 km from Bahir Dar city, the capital of Amhara Regional State. It has an altitude of 12.360 N, 37.280 E and a longitude of 12.60 N, 37.467 E with an elevation of 2,133 m above sea level, and is divided into 6 administrative areas (sub-cities) that consist of 21 kebeles (the smallest administrative units in Ethiopia). Gondar is among the most ancient and largely populated cities in the country. Currently, the city has an estimated population of 432,191; among these, 36,700 are preschool children. There is now one comprehensive specialized hospital and eight health centers providing health services to the population.

### Source and study population

All employed and unemployed mothers with children aged between 24 and 59 months, who lived in Gondar city, were used as the source population. The participants were available at their homes during the data collection period. Children who had anatomical or physical abnormalities were excluded from the study.

### Sample size and sampling procedure

The sample size was determined by using two population proportion formulas. The following premises were taken into account: confidence level = 95%, power (1-ß) = 84%, design effect = 2, ratio = 1:1, the proportion of stunting among under-five children of unemployed mothers is 39.5%, and the proportion of stunting among under-five children of employed women is 54.3%, taken from the previous study conducted at Adama town (14). Considering a design effect of 2 and a 10% non-response rate, a sample size of 385 was determined for both groups, implying a total of 770.

**Figure 1:**
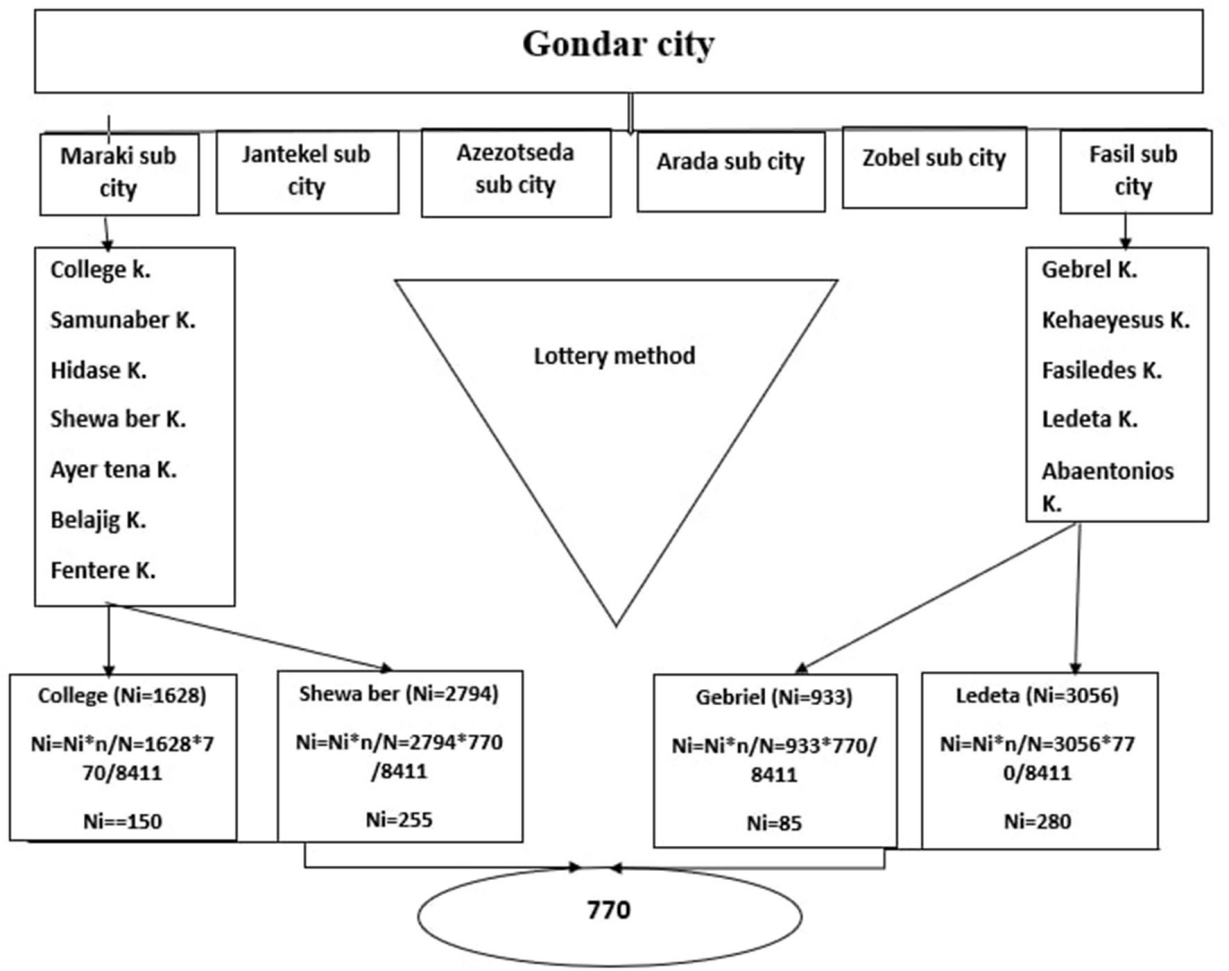
Schematic representation of sampling procedure for stunting of preschool children among employed and unemployed mothers residing in Gondar city in 2021.

To select study participants, multi-stage sampling followed by the systematic sampling technique was employed. Two sub-cities were randomly selected using a lottery method based on the WHO recommendation to include at least 20–30% of the total. Then, four kebeles (two kebeles from each subcity) were selected. The city health officers and the Mayor's Plan and Commission Office provided information on the total number of preschoolers in the chosen kebeles, and the total number of kids participating in the study was then proportionally distributed to each kebele using this information. Finally, systematic random sampling was utilized to choose the study participants from the list of children available to health extension workers in each kebele. If a family has more than one child between the ages of 24 and 59 months, one child was chosen at random using the lottery method (Figure 1).

### Data collection tool and procedure

A validated tool was taken from the literature (14, 17). An anthropometric measurement and a structured face-to-face interview were utilized to collect the data. Socio-demographic variables and other factors were gathered through face-to-face interviews with structured questionnaires that were created and translated into the local language through the literature of related research. Height measurements were taken from the child immediately following the interview with the child's mother. Using a handheld audiometer with a precision of 0.1 cm, standing height measurements were taken. To produce an accurate result, measurements were made three times, with the average being recorded. Using WHO Anthro software, anthropometry was converted to sex- and age-specific Z-scores to produce nutritional status indices (height for age).

According to the most recent WHO recommendations, dietary diversity is defined as children consuming the eight indicated food groups in a 24-h period. Breast milk, cereals, roots, tubers, legumes, nuts, dairy products, flesh foods (meats, fish, and poultry), eggs, and different fruits and vegetables are included in this list of food groups. Each of the eight food groups that are consumed is worth one point toward the overall dietary diversity score, which ranges from 0 to 8. Children who received a score of 5 for dietary diversity were categorized as having good dietary diversity, while those who received a score of 5 were categorized as having poor dietary diversity.

Based on the information gathered through the Household Questionnaire, the wealth index is generated. This questionnaire asks about the household's possession of various consumer goods, including a television and a car, as well as details about the home, such as the type of flooring, the type of drinking water source, the restrooms, and other elements that are indicative of financial position. Each asset owned by a household for which data are gathered is given a weight or factor score generated using principal component analysis. The generated asset scores are normalized using a normal distribution with a mean of zero and a standard deviation of 1. The breakpoints that distinguish three levels of financial status—lowest, middle, and highest—are then developed using these standardized scores.

### Data quality assurance

To ensure uniformity, the questionnaire was first written in English and then translated into Amharic and then back into English. It was reviewed and validated by nutritionists to check its appropriateness for assessing stunting. Prior to the period for data collection, the questionnaires were tested at kebele 1 (Belko), which is not part of the study, on 5% of the sample size. Amendments to the instrument, such as the clarification of unclear questions and ambiguous words, were made accordingly. Data collectors and supervisors were recruited based on their experience in data collection, and 1-day training was given on the objective of the study, the instrument, and the data collection procedures by the principal investigator. Furthermore, the tools were reviewed by four experts (two clinical nurses and two nurse academics). Daily checks for the completion of the filed questionnaires were made to assure the accuracy of the data.

### Data processing and analyzing

Statistical significance and strength of association were determined using crude and adjusted odds ratios with their 95% confidence intervals. In the measurement of association, a binary logistic regression model was fitted while assessing the association between the different variables and the dependent variable. First, each relationship between each independent variable and outcome variable was investigated using a univariable analysis. Those independent variables with a *p*-value of < 0.2 at the univariable analysis were included in a multivariable analysis to control potential confounding factors. After adjusting their effect on the outcome variable, those variables with a *p*-value of <0.05 with a 95% confidence that did not include null or zero values were recorded as factors significantly associated with stunting perspectives (overall stunting, stunting of children among employed mothers, and stunting of children among unemployed mothers).

## Results

### Socio-demographic characteristics

The study included 770 mother–child pairs (385 employed mothers and 385 jobless mothers), with a 100% response rate. The majority of mothers were married (92.6%), and among unmarried women, 10.39% and 4.4% were employed and unemployed, respectively (Table 1). More than half of mothers (66.8%) were aged 25–34 years. Overall, approximately 36.5% of mothers attend higher education, with one-fifth (20.0%) being unemployed mothers and more than half (53.5%) being employed mothers. Of all participants, 12.4% of mothers had a home servant; among this, one-fourth (24.4%) were employed mothers, and less than one-tenth (8%) were unemployed mothers.

**Table 1:** Stunting disparities and their associated factors among employed and unemployed mothers of preschool children in Gondar city: a comparative community-based cross-sectional study, 2021 (*n* = 770).

**Variable**	**Response**	**Employed (*n* = 385)**	**Unemployed (*n* = 385)**
		**Frequency %**	**Frequency %**
Maternal age	15–24	55 14.3	60 15.6
	25–34	261 67.8	254 65.9
	≥35	69 17.9	71 18.4
Marital status	Married	345 89.6	368 95.6
	Unmarried	40 10.4	17 4.4
Maternal education status	No education	24 6.2	70 18.2
	Primary	67 17.4	113 39
	Secondary	88 22.8	125 32.5
	College	206 53.5	77 20
Family size	≤ 4 5–6 ≥7	199 51.7 133 34.5 53 13.7	122 57.7 54 33.5 34 8.8
No under-five children	1	201 52.2	251 65.2
	2 and above	184 47.8	134 34.8
Wealth status	Poor	119 30.9	161 41.8
	Middle	120 31.2	114 29.6
	Rich	146 37.9	110 28.6
Home servant	Yes	94 24.4	31 8.0
	No	291 75.6	354 91.9

### Child and nutritional-related characteristics

Out of 770 preschool children who participated in the study, more than half (53.77%) were male children among employed mothers (Table 2). Among unemployed mothers, 50.13% were male children and 49.87% were female children. The median age of the children was 38 months, with a SD of 11. More than half (50.91%) of preschool children among employed mothers were aged 24–36, and 43.38 were aged 24–36 among unemployed mothers. Almost all (98.70%) of preschool children were fully vaccinated by both employed and unemployed mothers.

**Table 2:** Child and nutrition-related characteristics of preschool children living with employed and unemployed mothers in Gondar city in 2021 (*n* = 770).

**Variable**	**Response**	**Employed (*n* = 385)**	**Unemployed (n = 385)**
		**Frequency %**	**Frequency %**
Child age	24–36	196 50.9	167 43.4
	37–48	122 31.4	132 34.3
	49–59	67 17.4	86 22.3
Child sex	Male	207 53.7	193 50.1
	Female	178 46.2	192 49.8
Birth order	First	195 50.6	178 46.2
	Second	153 39.7	157 40.8
	Third and above	37 9.61	50 12.9
Immunization status	Fully vaccinated	380 98.70	380 98.7
	Not fully vaccinated	5 1.3	5 1.3
Vitamin A supplementation	Yes	328 85.2	322 83.6
	No	57 14.8	63 16.4
Dietary diversity	Poor	224 58.2	241 62.6
	Good	161 41.8	144 37.4

### Maternal-related characteristics

Almost all (97.92%) employed mothers and 95.84% of unemployed mothers had ANC visits (Table 3). Approximately 98.18% of employed mothers and 96.36% of unemployed women delivered their children in a health institution. More than three-quarters (78.18%) of employed mothers and exactly two-thirds (66.23%) of unemployed mothers received nutritional information. The majority of sources of nutritional information were health extension workers (64.71%, 50.17%) for unemployed and employed mothers, respectively. More than three-quarters (78.96%) of employed mothers and two-thirds (65.71%) of unemployed mothers were aged above 20 when they delivered their first child. One-quarter (25.19%) of employed mothers and below one-fifth (18.44%) of unemployed mothers were sick during their pregnancy.

**Table 3:** Maternal-related characteristics of employed and unemployed mothers living in Gondar city in 2021 (*n* = 770).

**Variable**	**Response**	**Employed (n = 385)**	**Unemployed (n = 385)**
		**Frequency %**	**Frequency %**
Maternal age at first birth	< = 19	81 21	132 34.3
	> = 20	304 78.9	253 65.7
ANC visit	Yes	377 97.9	369 95.8
	No	8 2.0	16 4.2
PNC visit	Yes	378 98.1	376 97.6
	No	7 1.8	9 2.3
Sickness during pregnancy	Yes	97 25.2	71 18.4
	No	288 74.8	314 81.6
Place of delivery	Health institution	378 98.2	371 96.4
	Home	7 1.8	14 3.64
Nutritional information	Yes	301 78.2	255 66.2
	No	84 21.8	130 33.7
Source of nutritional information	On mass media	117 38.8	66 25.8
	From health edu.	151 50.2	165 64.7
	Others	33 10.9	24 9.4

ANC, antenatal care; PNC, postnatal care.

### The overall magnitude of stunting

**Figure 2:**
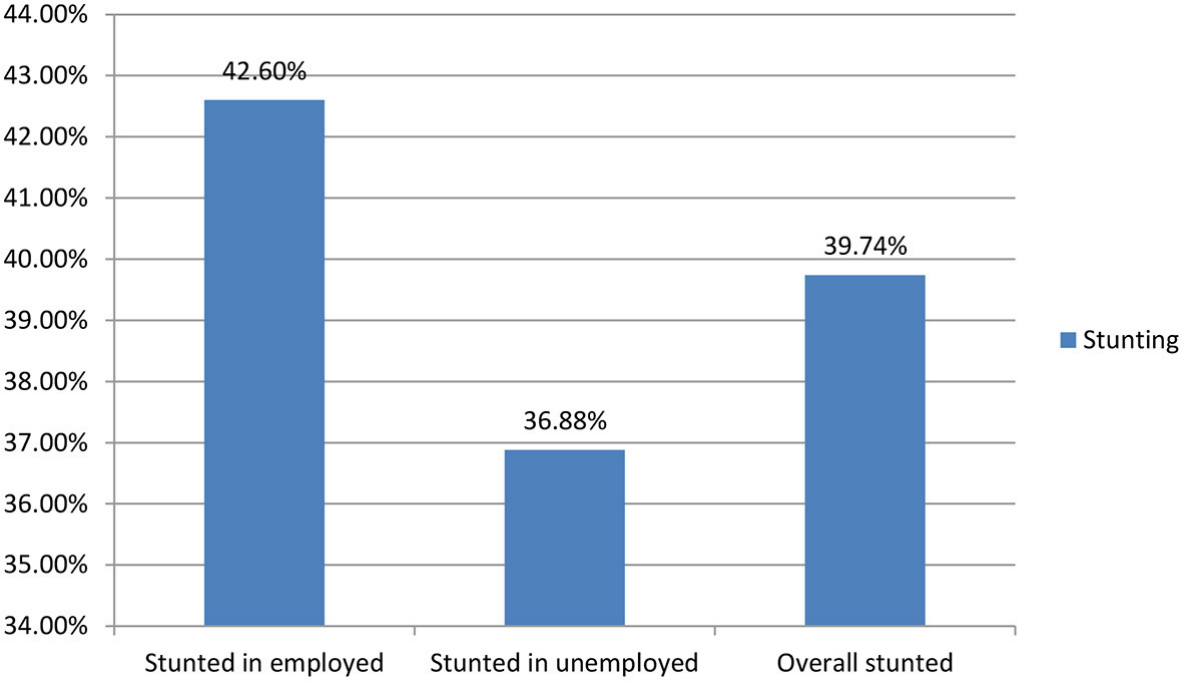
Prevalence of stunting among preschool children with employed and unemployed mothers in Gondar city in 2021.

The overall magnitude of stunting among preschool children in Gondar city was 39.74% (95% CI: 36.28–43.20). It was higher among male children (41.25%) than female children (38.11%). The magnitude of stunting among preschool children was higher among employed mothers than unemployed mothers (Figure 2).

### Comparisons of stunting in preschool children among employed and unemployed mothers

The magnitude of stunting was higher among preschool children of employed mothers (42.6%) (95% CI: 37.64–47.55) than among unemployed mothers (36.78% (95% CI: 32.04–41.72). Regarding socio-demographic factors, the magnitude of stunting was higher among male (59.14%) children of employed mothers compared to children of unemployed mothers (47.88%). The magnitude of stunting was higher among poor wealth status (55.63%) of unemployed mothers compared to poor wealth status (36.58) of employed mothers. Regarding child age, the magnitude of stunting was higher among children of employed mothers aged 24–36 (53.65%) than among children of unemployed mothers (40.14%).

### Factors associated with stunting of preschool children among employed mothers

A univariable analysis was carried out, and nine variables were associated with stunting among children of employed mothers. In a multivariable analysis, only five variables were found to be significantly associated with the stunting of preschool children among employed mothers. Children having mothers aged 15–24 were 4.59 times more likely to be stunted as compared to children having mothers aged more than 35 years [AOR = 4.59, 95% CI: 1.60–13.14], and children having family sizes of 7 and more were seven times more likely to be stunted as compared to children having family sizes of less than four [AOR = 7.12, 95% CI: 2.94–17.54] (Table 4). The presence of two or more children of under-five age was 1.92 times more likely to be stunted as compared to the presence of one under-five child in the family [AOR = 1.92, 95% CI: 0.3–0.89]. Preschool children who had a home servant in the family were 87.4% less likely to be stunted as compared to preschool children who had no home servant in the family [AOR = 0.126, 95% CI: 0.06–0.26].

**Table 4:** Univariable and multivariable logistic regression analyses of factors associated with stunting of preschool children among employed mothers in Gondar city in 2021.

**Variable**	**Response**	**Stunting**		**COR 95% CI**	**AOR 95% CI**	***P*-value**
		**No**	**Yes**			
Maternal age	15–24	20	35	**2.56 (1.23–5.3)**	**4.59 (1.6–13.14)**	**0.004**
	25–34	160	101	0.92 (0.54–1.6)	1.77 (0.83–3.780)	0.137
	≥35	41	28	1.0	1.0	
Child sex	Male	110	97	1.46 (0.97–2.19)	1.35 (0.82–2.18)	0.253
	Female	111	67	1.0	1.0	
Maternal education	No education	12	12	1.67 (0.71–3.91)	1.52 (0.52–4.40)	0.442
	Elementary	34	33	1.62 (0.93–2.83)	1.20 (0.58–2.47)	0.620
	Secondary	46	42	1.52 (0.92–2.53)	1.41 (0.74–2.7)	0.294
	College and above	14	77	1.0	1.0	
Family size	≤ 4	127	72	1.0	1.0	
	5–6	81	52	1.13 (0.72–1.78)	1.52 (0.83–2.80)	0.177
	≥7	13	40	**5.43 (2.72–10.8)**	**7.19 (2.95–17.55)**	**0.001**
Under-five children	1	129	72	1.0	1.0	
	2 and more	92	92	**1.79 (1.19–2.69)**	**1.92 (1.12–3.29)**	**0.017**
Home servant	Yes	81	13	**0.15 (0.08–0.28)**	**0.126 (0.06–0.26)**	**0.001**
	No	140	151	1.0	1.0	
Nutritional info	Yes	180	121	0.64 (0.39–1.04)	0.84 (0.46–1.55)	0.597
	No	41	43	1.0	1.0	
Maternal sickness	Yes	47	50	**1.62 (1.022–2.58)** 1.0	**1.8 (1.04–3.15)** 1.0	**0.037**
	No	174	114			
Birth order	First	118	77	1.0	1.0	
	Second	82	71	1.33 (0.86–2.03)	1.55 (0.83–2.86)	0.162
	Third and above	21	16	1.17 (0.57–2.37)	0.82 (0.29–2.29)	0.712
Child age	24–36	108	88	1.0	1.0	
	37–48	66	56	1.04 (0.66–1.64)	1.07 (0.61–1.89)	0.799
	49–59	47	20	0.52 (0.28–0.95)	0.65 (0.31–1.34)	0.252
Dietary diversity	Poor	117	107	1.66 (1.10–2.53)	1.59 (0.97–2.6)	0.065
	Good	104	57	1.0	1.0	
Wealth status	Poor	59	60	1.23 (0.76–2.00)	0.63 (0.33–1.20)	0.163
	Middle	82	38	0.56 (0.34–0.93)	0.60 (0.32–1.14)	0.121
	Rich	80	66	1.0	1.0	

AOR, adjusted odds ratio; COR, crude odds ratio.

### Factors associated with stunting of preschool children among unemployed mothers

A univariable analysis was carried out to analyze the factors associated with stunting in preschool children among unemployed mothers. Seven variables were significantly associated with stunting: maternal age, maternal education, home servant, sickness during pregnancy, child age, vitamin A status, and wealth status. In a multivariable analysis, only two factors were significantly associated with stunting among children of unemployed mothers. Children having mothers aged 15–24 were 2.8 times more likely to be stunted as compared to children having mothers aged more than 35 years [AOR = 2.8, 95% CI: 1.26–6.34] (Table 5). Children having mothers with middle wealth status were 32% less likely to be stunted as compared to children having mothers with poor wealth status [AOR = 0.32; 95% CI: 0.18–0.57].

**Table 5:** Univariable and multivariable logistic regression analyses of factors associated with stunting of preschool children among unemployed mothers in Gondar city in 2021.

**Variables**	**Response**	**Stunting**		**COR (95% CI)**	**(AOR 95% CI)**	***P*-value**
		**Yes**	**No**			
Unemployed mother sage	15–24	34	26	2.56 (1.26–5.20)	**2.83 (1.26–6.34)**	**0.011**
	25–34	84	170	0.96 (0.55–1.68)	1.13 (.60–2.11)	0.701
	≥35	24	47	1.0	1.0	
Unemployed mother education	No education	30	40	1.56 (0.79–3.0)	1.49 (0.70–3.16)	0.336
	Elementary	44	69	1.32 (0.72–2.4)	1.07 (0.55–2.07)	0.835
	Secondary	43	82	1.09 (0.59–1.99)	1.004 (0.52–1.91)	0.990
	College and above	25	52	1.0	1.0	
Presence of a home servant	Yes	8	23	0.57 (0.25–1.31)	0.7 (0.29–1.72)	0.457
	No	134	220	1.0	1.0	
Sickness during pregnancy	Yes	33	38	1.0	1.0	
	No	109	205	0.61 (0.36–1.03)	0.7 (0.29–1.72)	0.457
Child age in months	24–36	57	110	1.0	1.0	
	37–48	59	73	1.56 (0.97–2.44)	1.55 (0.93–2.58)	0.086
	49–59	26	60	0.83 (0.47–1.46)	0.89 (0.49–1.63)	0.722
Vitamin A supplementation	Yes	142	198	1.0	1.0	
	No	18	45	0.64 (0.35–1.15)	0.63 (0.33–1.58)	0.153
Unemployed mother Wealth status	Poor	79	82	1.0	1.0	
	Medium	25	89	**0.29 (0.17–0.50)**	**0.32 (0.18–0.57)**	**0.001**
	Rich	38	72	**0.54 (0.33–0.90)**	0.66 (0.39–1.14)	0.142

### Factors associated with overall stunting of preschool children

In a univariable analysis, 14 variables were associated with overall stunting among preschool children. In a multivariable analysis, only seven variables were associated with overall stunting among preschool children. Children from employed mothers were 1.5 times more likely to be stunted as compared to children from unemployed mothers [AOR = 1.54, 95% CI: 1.08–2.18] (Table 6). Preschool children whose mothers were aged 15–24 were four times more likely to be stunted as compared to mothers aged above 35 years [AOR = 4.31; 95% CI: 2.1–8.54]. Preschool children in a family of 7 and above were 3.4 times more likely to be stunted as compared to children living in a family of less than 4 [AOR = 3.39, 95% CI: 1.88–6.14]. Children from mothers who have no home servant or caregiver were 4.5 times more likely to be stunted as compared to children with caregivers [AOR = 4.49, 95% CI: 1.88–6.14]. Children with poor dietary diversity were 1.5 times more likely to be stunted as compared to children with good dietary diversity [AOR = 1.49, 95% CI: 1.06–2.09]. Children in families with middle wealth status were 48% less likely to be stunted as compared to children whose families had poor wealth status [AOR = 0.48.95 CI:0.31–0.74].

**Table 6:** Univariable and multivariable logistic regression analyses of overall factors associated with overall stunting of preschool children in Gondar city in 2021.

**Variables**	**Response**	**Stunting**		**COR (95% CI)**	**AOR (95% CI)**	***P*-value**
		**Yes**	**No**			
Child sex	Male	165	235	1.14 (0.85–1.52)	1.04 (0.75–1.4)	0.225
	Female	141	229	1.0	1.0	
Maternal age	15–24	69	46	**2.53 (1.53–4.21)**	**4.3 (2.17–8.54)**	**0.001**
	25–34	185	330	0.94 (0.64–1.40)	1.38 (0.83–2.26)	0.201
	≥35	52	88	1.0	1.0	1.000
Maternal education	No education	42	103	1.43 (0.89–2.3)	1.23 (0.67–2.24	0.451
	Elementary	77	128	1.32 (0.90–1.94)	0.94 (0.58–1.5)	0.812
	Secondary	85	128	1.17 (0.82–1.70)	1.05 (0.68–1.64)	0.811
	College and above	102	181	1.0	1.0	
Employment	Employed	164	221	1.27 (0.95–1.69)	**1.54 (1.08–2.18)**	**0.015**
	Unemployed	142	243	1.0	1.0	
Family size No of under five	≤ 4	149	272	1.0	1.0	
	5–6	103	159	1.18 (0.86–1.62)	**1.5 (1.004–2.29)**	**0.048**
	≥7	103	159	**2.98 (1.85–4.80)**	**1.88–6.15**	**0.000**
	1	163	289	1.0	1.0	
	2 and above	143	175	**1.45 (1.08–1.94)**	**1.46 (1.03–2.078)**	**0.033**
Home servant	Yes	21	104	1.0	1.0	
	No	285	360	**3.92 (2.39–6.42)**	**4.49 (2.58–7.82)**	**0.001**
Child age	24–36	145	218	1.0	1.0	
	37–48	115	139	1.24 (0.89–1.72)	1.35 (0.94–1.95)	0.153
	49–59	46	107	**0.64 (0.43–0.96)**	0.78 (0.49–1.23)	0.283
Birth order	First	139	234	1.0	1.0	
	Second	135	175	1.29 (0.95–1.76)	1.46 (0.97–2.18)	0.066
	3 and above	32	55	0.98 (0.6–1.58)	0.93 (0.47–1.8)	0.843
Sickness during Px.	Yes	83	85	**1.65 (1.17–2.34**)	1.47 (0.99–2.16)	0.052
	No	223	379	1.0	1.0	
Vitamin A status	Yes	267	383	1.0	1.0	
	No	39	81	0.69 (0.45–1.04)	0.66 (0.42–1.05)	0.081
Nutritional information	Yes	21	345	1.0	1.0	
	No	95	119	0.69 (0.45–1.04)	1.07 (0.74–1.55)	0.712
Dietary diversity	Poor	200	265	**1.41 (1.05–1.9)**	**1.49 (1.06–2.09)**	**0.019**
	Good	106	199	1.0	1.0	
Wealth status	Poor	139	141	1.0	1.0	
	Medium	63	171	**0.37 (0.26–0.54)**	**0.48 (0.32–0.73)**	**0.001**
	Rich	104	152	**0.69 (0.49–0.98)**	0.86 (0.57–1.3)	0.489

Model fitness test: Hosmer and Lemeshow test = 0.197.

## Discussion

This study disclosed the prevalence and associated factors of stunting among preschool children of employed and unemployed mothers. Accordingly, the overall prevalence of stunting was 39.7% (95 % CI: 36.3–43.2). The prevalence of stunting in preschool children was 42.59% (95% CI: 37.64–47.55) among employed mothers and 36.88 % [95% CI: (32.04–41.72)] among unemployed mothers which shows there was a significant difference in the prevalence of stunting among preschool children of employed and unemployed mothers. The overall prevalence of stunting among preschool children was comparable with the finding from Albuko district South Wollo Zone, the overall prevalence of stunting was 39.3% (18) (95% CI: 36.3%−42.3%), Adama 39.5% (14), Bahir Dar 38% (19). The similar prevalence might be such as socio-demographic and economic characteristics (20) and worldwide comparable with studies conducted in Pakistan 38% (21) and Mali 38.3% (22). This similar finding might be due to the fact that these countries are at the same level of development as our country since they are developing countries.

The overall prevalence of stunting in this study was lower than in the previous study conducted in Ethiopia: Hawassa (53.4%) (23) and Arba Minch (47.1%) (24). This disparity could be attributed to agroecological and socio-demographic differences, as well as the inclusion of rural kebeles in the study in Arba Minch, where stunting is more prevalent than in urban settlements (25, 26).

The overall prevalence of stunting in this age group (2–5 years) was lower in this study than in previous studies conducted in Africa, Burundi (57.7%), Malawi (47.1%), and Zambia (22). This difference might be due to socioeconomic, cultural, and childcare practices among countries.

In this study, the overall frequency of stunting was higher than in previous studies in Ethiopia: Jimma, 21.8%% (27), India, 20% (28), and Southern Iran, 18% (29). The higher prevalence of stunting among children in this study than in previous findings in Ethiopia could be due to differences in socioeconomic, cultural, food habits, environmental conditions, and public service consumption of the people in the research area between the southern and northern parts of Ethiopia (30). On the other hand, the higher prevalence of stunting among children in this study than previous findings in India and Southern Iran could be due to differences in socioeconomic status and cultural differences across countries; moreover, the economic and health policies of India and Iran are better compared to Ethiopia.

The overall prevalence of this study was also higher than studies in Africa; Ghana 28.2% (31), Nigeria, 21.4%, and Uganda 29% (32). The disparity could be attributed to socioeconomic, cultural, and lifestyle differences between the two countries. This discrepancy could also be related to differences in childcare practices between countries.

The prevalence of stunting in this study shows a significant difference between preschool children stunting among employed and unemployed moms, with employed mothers having a greater prevalence of stunting than unemployed mothers. This finding is corroborated by the findings of investigations conducted in many parts of the world: According to Sri Lanka and Iran, the children of unemployed mothers were substantially taller than the children of employed mothers (*p*-value<0.05) (33, 34). In India, children with employed moms had a stunting of 11.5%, while children with unemployed mothers had a stunting rate of 5.5% (35). Another study conducted in India supports our findings, revealing that children of unemployed moms were substantially taller than children of employed mothers (36). On the one hand, the discrepancy could be explained by the fact that unemployed moms are more likely to look after their babies than employed mothers, have time to make nutritious food, and take them to healthcare providers on a regular basis (11). On the other hand, working mothers who lack the time during the day to care for their children use nurseries. Children in nurseries experience increased exposure to a range of illnesses as well as decreased attention to their psychological and physical needs, which results in stunting (34), and employed mothers' workload prevents breastfeeding practices (37). In addition to breastfeeding practices, the working status of mothers also shortens the duration of breastfeeding due to employment rules and regulations, such as less maternity leave (3 months in the Ethiopian context), and employed mothers have less opportunity to stay at home, compromising breastfeeding, and lack a private space for child feeding at the workplace (38). Moreover, in Gondar town, 86.5% of households live in destitution. A very small asset portfolio, multiple enhanced vulnerabilities, and an unsupportive and hindering institutional setting define the residents' livelihood context. The consequence of the interaction of these elements is poverty and insecure livelihoods (39); as a result, these variables exacerbate child stunting. This study's findings were similarly consistent with those of an Ethiopian study, which found that unemployed women were 23% less likely [AOR: 0.768; 95% CI (0.646–0.912), *p* = 0.003] to have a stunted child than employed moms (40), Another study in Ethiopia found that children with merchant mothers were more likely to suffer stunting than children with housewife mothers (41). Possible explanations for this disparity are that unemployed mothers have time to prepare nutrition-rich food and take their children to healthcare providers on a regular basis, whereas in developing countries such as Ethiopia, wealthier or “better-off” men usually do not want their wives to work, so their wives provide appropriate care and nutrition for their children (40).

The findings of this study differed from those of a study conducted in Ethiopia by the Wolaita Zone Administration, which discovered that the magnitude of stunting among children of employed and unemployed moms was 18.5% and 26.5%, respectively (17). This disparity may be attributable to the fact that our study was conducted in an urban area, which had a higher proportion of employed moms than in rural areas such as the Wolaita Zone (17, 42).

Stunting in preschool children of employed mothers is caused by a variety of circumstances. In this aspect, having no house servant increases the likelihood of stunting eight times more than having a home servant (AOR = 7.9, 95% CI = 3.76, 16.75), *p*-value = 0.001). This finding is consistent with a study conducted in the Wolaita Zone Administration, which found that mothers who did not have a home servant had approximately three times the odds of stunting as their counterparts (AOR = 2.58, 95% CI = (1.35, 4.91), *P*-value = 0.004) (17). This could be explained as the presence of a home servant can handle childcare issues in the absence of a mother, which can contribute significantly to the child's nutritional status when compared to employed mothers who are responsible for caring for the child in addition to routine governmental duties (17). Another explanation for stunting in preschool children of employed mothers is the lack of family support and a changing composition of the workforce, especially women's increased participation rates in informal and heavy work conditions in Ethiopia, including Gondar, which has made it a great challenge to find ways for breastfeeding to prevent stunting (43).

Regarding the associated factors of stunting among unemployed mothers, maternal age <24 years had higher odds of stunting when compared to maternal age >35 (AOR = 2.83, 95% CI: 1.26–6.34). This finding is consistent with the finding from Uganda (44) in which mothers aged 15–24 years had 30% higher odds of stunting compared to mothers aged 35–49 years (*p*-value 0.04) (44). A possible explanation for the better nutritional outcomes among children of older women is that older mothers may have greater experience in childcare than younger mothers. Other young mothers might have limited access to socioeconomic resources to meet their children's dietary demands (45).

Another factor associated with preschool child stunting among unemployed moms was medium wealth status, which reduced the risk of stunting by 68% when compared to poor wealth status. This study was very similar to the one conducted in Labella, Ethiopia (46).

The current study discovered that bigger family sizes and the presence of more than two children under the age of five were positively associated with stunting. This finding is consistent with a study conducted in Pakistan (21), Brazil (47), and Ethiopia (18) in which children in families with seven or more family members were more likely to be stunted than those in families with two to four family members. Similarly, children in households with two or more under-five children were more likely to develop stunting than those in households with only one under-five child. This could be related to resource depletion, which exposes people to poverty and decreases food availability, as well as affecting household food consumption patterns by driving them to buy low-quality food, skip meals, and rely on a repetitive diet (16, 41).

According to this finding, stunting of preschool children among both employed and unemployed mothers with a lower wealth index was associated with stunting. This finding is similar to those found in studies conducted in Ethiopia, Dabat HDSS (48, 49), Bangladesh (50), and Nepal (51). This could be explained by the fact that more money promotes dietary diversity, which in turn improves nutrient adequacy and frequency as well as nutritional status.

This study discovered that a lack of dietary diversity is linked to stunting; this finding is consistent with a Bangladeshi study (52), as well as other studies in Ethiopia (18, 20), in which a low diet diversity score (DDS) of <4 was statistically significant with child stunting. This could be due to inadequate complementary feeding and a general lack of critical nutrients in addition to pure calorie intake, which is one reason for stunted growth. To prevent stunting, children must be fed meals that meet the minimum standards (four) for diet diversification.

### Limitation

There might be a possibility of recall bias and social desirability bias by mothers on variables such as ANC, PNC visit, and nutritional information.

## Conclusion

Preschool children of employed mothers had a higher prevalence of stunting than preschool children of unemployed mothers. Maternal age, household size, number of children under the age of five, availability of a housekeeper, and inadequate dietary diversity were all associated with stunting in preschool children of employed moms. Stunting in preschool children of unemployed mothers was connected with maternal age and wealth level.

The nutrition intervention should focus on encouraging dietary diversity to combat the existing nutrition-associated stunting in children. Similarly, further research on the difference between employed and unemployed mothers' child stunting status and investigation on the extra variables such as the number of hours worked by an employed mother is also recommended to upcoming researchers.

## Data availability statement

The original contributions presented in the study are included in the article/supplementary files, further inquiries can be directed to the corresponding author.

## Ethics statement

The studies involving humans were approved by the School of Nursing College of Medicine and Health Science (Ref.no.-C/H/N164/7/2013) on behalf of the Research Ethical Committee of University of Gondar. The studies were conducted in accordance with the local legislation and institutional requirements. All methods were carried out in accordance with ethical and human rights standards, and with relevant guidelines and regulations. Written informed consent for participation in this study was provided by the participants' legal guardians/next of kin. Confidentiality was kept by using codes rather than using their names. They were informed initially about their right to withdraw from the study at any time during the interview process. When they were informed about their right to withdraw, they agreed to leave the study whenever they are not feeling comfortable. At the end of the interview and anthropometry measurements, children with stunting were linked to malnutrition centers.

## Author contributions

BT conceived the idea, wrote the manuscript, participated in the data collection process, analyzed the data, and drafted the manuscript. BTT approved the proposal with some versions, participated in data analysis, and reviewed the manuscript. AA contributed to analysis, interpretation, reporting, and manuscript writing. AZ conceptualized the study and was involved in design, analysis, interpretation, reporting, and manuscript writing. All authors approved the final draft of the manuscript.

## References

[1] Organization WHO. WHO Child Growth Standards: Length/Height-for-Age, Weight-for-Age, Weight-for-Length, Weight-for-Height and Body Mass Index-for-Age: Methods and Development. World Health Organization (2006).

[2] Organization WHO. UNICEF/WHO/The World Bank Group Joint Child Malnutrition Estimates: Levels and Trends in Child Malnutrition: Key Findings of the 2020 Edition. (2020).

[3] Black RE Victora CG Walker SP Bhutta ZA Christian P De Onis M. Maternal and child undernutrition and overweight in low-income and middle-income countries. Lancet. (2013) 382:427–51. 10.1016/S0140-6736(13)60937-X 23746772

[4] Mini Demographic and Health Survey Key Indicators. EPHI and ICF (2019).

[5] Derso T Tariku A Biks GA Wassie MM. Stunting, wasting and associated factors among children aged 6–24 months in Dabat health and demographic surveillance system site: a community based cross-sectional study in Ethiopia. BMC Pediatr. (2017) 17:1–9. 10.1186/s12887-017-0848-2 28376746 PMC5379504

[6] Glewwe P King EM. The impact of early childhood nutritional status on cognitive development: does the timing of malnutrition matter? World Bank Econ Rev. (2001) 15:81–113. 10.1093/wber/15.1.81

[7] Ekholuenetale M Barrow A Ekholuenetale CE Tudeme G. Impact of stunting on early childhood cognitive development in Benin: evidence from demographic and health survey. Egypt Pediat Assoc Gazette. (2020) 68:1–11. 10.1186/s43054-020-00043-x

[8] Stevens GA Finucane MM Paciorek CJ Flaxman SR White RA Donner AJ. Trends in mild, moderate, and severe stunting and underweight, and progress towards MDG 1 in 141 developing countries: a systematic analysis of population representative data. Lancet. (2012) 380:824–34. 10.1016/S0140-6736(12)60647-3 22770478 PMC3443900

[9] Angela K Thorne-Lyman AL Manohar S Shrestha B Klemm R Adhikari RK. Preschool child nutritional status in Nepal in 2016: a national profile and 40-year comparative trend. Food Nutr Bull. (2020) 41:152–66. 10.1177/0379572120916343 32522131

[10] Rajpal S Kim R Joe W Subramanian S. Stunting among preschool children in india: temporal analysis of age-specific wealth inequalities. Int J Environ Res Public Health. (2020) 17:4702. 10.3390/ijerph17134702 32629904 PMC7370207

[11] Rashad AS Sharaf MF. Does maternal employment affect child nutrition status? New evidence from Egypt Oxford. Develop Stud. (2019) 47:48–62. 10.1080/13600818.2018.1497589

[12] Abreha SK Walelign SZ Zereyesus YA. Associations between women's empowerment and children's health status in Ethiopia. PLoS ONE. (2020) 15:e0235825. 10.1371/journal.pone.0235825 32687506 PMC7371184

[13] Brauner-Otto S Baird S Ghimire D. Maternal employment and child health in Nepal: the importance of job type and timing across the child's first five years. Soc Sci Med. (2019) 224:94–105. 10.1016/j.socscimed.2019.02.009 30771663 PMC6532054

[14] Wondafrash M Admassu B Bayissa Z Geremew F. Comparative study on nutritional status of under five children with employment status of mothers in Adama Town, Central Ethiopia. Matern Pediatr Nutr. (2017) 3:117. 10.4172/2472-1182.1000117

[15] Kennedy E Tessema M Hailu T Zerfu D Belay A Ayana G. Multisector nutrition program governance and implementation in Ethiopia: opportunities and challenges. Food Nutr Bull. (2015) 36:534–48. 10.1177/0379572115611768 26531747

[16] Tariku A Woldie H Fekadu A Adane AA Ferede AT Yitayew S. Nearly half of preschool children are stunted in Dembia district, Northwest Ethiopia: a community based cross-sectional study. Arch Public Health. (2016) 74:13. 10.1186/s13690-016-0126-z 27092252 PMC4834824

[17] Zewdu D Halala Handiso Y. Under-nutrition of 2–5 years old children and associated factor among employed and unemployed women: comparative cross-sectional study. Cogent Food Agricult. (2020) 6:1801215. 10.1080/23311932.2020.1801215

[18] Berhanu G Mekonnen S Sisay M. Prevalence of stunting and associated factors among preschool children: a community based comparative cross sectional study in Ethiopia. BMC Nutrition. (2018) 4:1–15. 10.1186/s40795-018-0236-9 32153889 PMC7050938

[19] Jalata BR. Nutritional status and associated factors among preschool children in Bahir Dar City Administration, Northern Ethiopia: a cross-sectional study. J Food Nutri Sci. (2020) 8:43–54. 10.11648/j.jfns.20200803.11

[20] Girma A Woldie H Mekonnen FA Gonete KA Sisay M. Undernutrition and associated factors among urban children aged 24–59 months in Northwest Ethiopia: a community based cross sectional study. BMC Pediatr. (2019) 19:1–11. 10.1186/s12887-019-1595-3 31255179 PMC6599324

[21] Naz L Patel KK Uzoma IE. The prevalence of undernutrition and associated factors among preschool children: evidence from Pakistan demographic and health survey 2017–18. Child Youth Serv Rev. (2020) 119:105579. 10.1016/j.childyouth.2020.105579

[22] Akombi BJ Agho KE Merom D Renzaho AM Hall JJ. Child malnutrition in sub-Saharan Africa: a meta-analysis of demographic and health surveys (2006-2016). PLoS ONE. (2017) 12:e0177338. 10.1371/journal.pone.0177338 28494007 PMC5426674

[23] Wolde T Belachew T Birhanu T. Prevalence of undernutrition and determinant factors among preschool children in Hawassa, Southern Ethiopia. Prevalence. (2014) 29:16–24.

[24] Bogale B Gutema BT Chisha Y. Prevalence of stunting and its associated factors among children of 6–59 Months in arba minch health and demographic surveillance site (HDSS), southern Ethiopia: a community-based cross-sectional study. J Environ Public Health. (2020) 2020:973. 10.1155/2020/9520973 32280353 PMC7115144

[25] Abdulahi A Shab-Bidar S Rezaei S Djafarian K. Nutritional status of under five children in Ethiopia: a systematic review and meta-analysis. Ethiop J Health Sci. (2017) 27:175–88. 10.4314/ejhs.v27i2.10 28579713 PMC5440832

[26] De Onis M Blössner M Borghi E. Prevalence and trends of stunting among pre-school children, 1990–2020. Public Health Nutr. (2012) 15:142–8. 10.1017/S1368980011001315 21752311

[27] Melese S Bedatu G Kalkidan H. Prevalence of undernutrition and associated factors among preschool children in Jimma town, South West Ethiopia. Af J Food Agricult Nutriti Develop. (2020) 20:15954–77. 10.18697/ajfand.91.18255

[28] Jayalakshmi R Kannan S. The catch-up growth in stunted children: analysis of first and second India human development survey data. Indian J Commun Med. (2019) 44:199. 10.4103/ijcm.IJCM_127_18 31602102 PMC6776956

[29] Fatemi MJ Dianatinasab M Sharifnia G Moravej H Fararouei M. Growth retardation among children in southern Iran: a 7-year population based cohort study. BMC Public Health. (2020) 20:1–9. 10.1186/s12889-020-09511-w 32917173 PMC7488575

[30] Geleta D Tesfaye N Zara M. Stunting and the Associated Factors among Under-five Children in Shire Endaslassie Town, Tigray, North West Ethiopia. (2021). 10.21203/rs.3.rs-470895/v1

[31] Ali Z Saaka M Adams A-G Kamwininaang SK Abizari A-R. The effect of maternal and child factors on stunting, wasting and underweight among preschool children in Northern Ghana. BMC nutrition. (2017) 3:31. 10.1186/s40795-017-0154-2 32153813 PMC7050753

[32] Mugarura D NINSIIMA HI wambuyi Kinyi H Eze DE Tumwesigire S Prossy V. High prevalence of stunting in preschool children (1-5years) attending selected health centers in a food rich Area-Bushenyi District Southwestern Uganda. Arch Public Health. (2021) 79:1–2. 10.21203/rs.3.rs-31913/v1 34336276 PMC8315884

[33] Galgamuwa LS Iddawela D Dharmaratne SD Galgamuwa G. Nutritional status and correlated socio-economic factors among preschool and school children in plantation communities, Sri Lanka. BMC Public Health. (2017) 17:1–11. 10.1186/s12889-017-4311-y 28464868 PMC5414369

[34] Fatemi MJ Fararouei M Moravej H Dianatinasab M. Stunting and its associated factors among 6–7-year-old children in southern Iran: a nested case–control study. Public Health Nutr. (2019) 22:55–62. 10.1017/S136898001800263X 30319086 PMC10260474

[35] Anuja A Sangeetha A Samuel Sundar Doss TJ. The effect of maternal employment on the nutritional status of pre-school children. Nat J Physiol Pharmacy Pharmacol. (2019) 9:1156–8. 10.5455/njppp.2019.9.082891009201923373036

[36] Yeleswarapu BK Nallapu SSR A. comparative study on the nutritional status of the pre-school children of the employed women and the unemployed women in the urban slums of Guntur. J Clin Diag Res JCDR. (2012) 6:1718. 10.7860/JCDR/2012/4395.2629 23373036 PMC3552212

[37] Chekol DA Biks GA Gelaw YA Melsew YA. Exclusive breastfeeding and mothers' employment status in Gondar town, Northwest Ethiopia: a comparative cross-sectional study. Int Breastfeed J. (2017) 12:1–9. 10.1186/s13006-017-0118-9 28638435 PMC5473972

[38] Thulier D Mercer J. Variables associated with breastfeeding duration. J Obst Gynecol Neon Nurs. (2009) 38:259–68. 10.1111/j.1552-6909.2009.01021.x 19538614

[39] Yirga B. The livelihood of urban poor households: A sustainable livelihood approach in urbanizing Ethiopia. The case of Gondar City, Amhara National State. Poverty Public Policy. (2021) 13:155–83. 10.1002/pop4.306

[40] Amaha ND Woldeamanuel BT. Maternal factors associated with moderate and severe stunting in Ethiopian children: analysis of some environmental factors based on 2016 demographic health survey. Nutr J. (2021) 20:1–9. 10.1186/s12937-021-00677-6 33639943 PMC7916293

[41] Fikadu T Assegid S Dube L. Factors associated with stunting among children of age 24 to 59 months in Meskan district, Gurage Zone, South Ethiopia: a case-control study. BMC Public Health. (2014) 14:800. 10.1186/1471-2458-14-800 25098836 PMC4131046

[42] Nebebe AF. Causes of Rural-Urban Migration and Employment Challenges in Urban Ethiopian.

[43] Skafida V. Juggling work and motherhood: the impact of employment and maternity leave on breastfeeding duration: a survival analysis on Growing Up in Scotland data. Matern Child Health J. (2012) 16:519–27. 10.1007/s10995-011-0743-7 21274609

[44] Nankinga O Kwagala B Walakira EJ. Maternal employment and child nutritional status in Uganda. PLoS ONE. (2019) 14:e0226720. 10.1371/journal.pone.0226720 31856209 PMC6922416

[45] Semali IA Tengia-Kessy A Mmbaga EJ Leyna G. Prevalence and determinants of stunting in under-five children in central Tanzania: remaining threats to achieving Millennium development goal 4. BMC Public Health. (2015) 15:1–6. 10.1186/s12889-015-2507-6 26590803 PMC4654796

[46] Yalew B Amsalu F Bikes D. Prevalence and factors associated with stunting, underweight and wasting: a community based cross sectional study among children age 6–59 months at Lalibela Town, Northern Ethiopia. J Nutr Disorders Ther. (2014) 4:2161. 10.4172/2161-0509.1000147

[47] Ferreira HdS Albuquerque GT Santos TRd Barbosa RdL Cavalcante AL Duarte LEC. Stunting and overweight among children in Northeast Brazil: prevalence, trends (1992-2005-2015) and associated risk factors from repeated cross-sectional surveys. BMC *Public Health*. (2020) 20:1–15. 10.1186/s12889-020-08869-1 32434581 PMC7238646

[48] Tariku A Biks GA Derso T Wassie MM Abebe SM. Stunting and its determinant factors among children aged 6–59 months in Ethiopia. Ital J Pediatr. (2017) 43:112. 10.1186/s13052-017-0433-1 29258578 PMC5735819

[49] Kassie GW Workie DL. Determinants of under-nutrition among children under five years of age in Ethiopia. BMC Public Health. (2020) 20:399. 10.1186/s12889-020-08539-2 32220224 PMC7099779

[50] Akram R Sultana M Ali N Sheikh N Sarker AR. Prevalence and determinants of stunting among preschool children and its urban–rural disparities in Bangladesh. Food Nutr Bull. (2018) 39:521–35. 10.1177/0379572118794770 30157677

[51] Budhathoki SS Bhandari A Gurung R Gurung A Ashish K. Stunting among under 5-year-olds in Nepal: trends and risk factors. Matern Child Health J. (2020) 24:39–47. 10.1007/s10995-019-02817-1 31776750 PMC7048700

[52] Rah JH Akhter N Semba RD De Pee S Bloem MW Campbell AA. Low dietary diversity is a predictor of child stunting in rural Bangladesh. Eur J Clin Nutr. (2010) 64:1393–8. 10.1038/ejcn.2010.171 20842167

